# Gastrin-releasing peptide is essential for generalization of auditory conditioned fear under stress

**DOI:** 10.1186/s13041-025-01214-w

**Published:** 2025-05-15

**Authors:** Yi Wu, Xiance Ni, Hisashi Mori, Ran Inoue

**Affiliations:** 1 Department of Molecular Neuroscience, Faculty of Medicine, University of Toyama, Toyama, 930-0194 Japan; 2 Graduate School of Innovative Life Science, University of Toyama, Toyama, Japan; 3 Research Center for Idling Brain Science, University of Toyama, Toyama, Japan

**Keywords:** Auditory fear generalization, Gastrin-releasing peptide, Acute restraint stress, Auditory cortex, Fear memory

## Abstract

Fear generalization, which allows animals to respond adaptively to cues similar to original threatening ones, is generally beneficial for survival. However, an inability to distinguish between threat and safety, leading to the overgeneralization of fear to non-threatening stimuli, is maladaptive and is implicated in anxiety disorders such as post-traumatic stress disorder (PTSD). The neuropeptide gastrin-releasing peptide (GRP) is known to modulate fear memory under stress, yet its role in response to intense aversive stimuli remains less understood. In this study, we used GRP knockout (*Grp*^−/−^) mice to examine the role of GRP in enhancing fear responses to conditioned stimulus (10 kHz tone, CS+) and non-conditioned stimulus (2 kHz tone, CS-) in a model of auditory fear conditioning with high-intensity footshocks following single acute restraint stress (RS). Our findings reveal that GRP is required not only for enhanced response to CS+ but also for generalized fear responses to CS-. Furthermore, we observed that infusion of GRP into the auditory cortex (AC) of *Grp*^−/−^ mice restores freezing behavior in response to CS- and fear generalization. Additionally, GRP in the AC is essential for the generalization of CS+ responsive neurons to respond to CS- during fear memory retrieval. These results highlight a novel role for GRP in the mechanisms underlying maladaptive fear in highly stressful situations, offering potential new targets for treating anxiety-related disorders.

**Supplementary Information:**

The online version contains supplementary material available at 10.1186/s13041-025-01214-w.

## Introduction

Properly controlled fear is essential for survival. When confronted with a potential threat, an animal must select an appropriate defensive response based on previous experiences, assessing cues and contextual information that may predict safety or danger. However, the aversive experiences are never completely identical, and threat cues may only partially resemble the original cue. Generalization allows responding to cues that are unlike the original cue [1]. Although the fear generalization may be generally adaptive, the inability to distinguish threat from safety and the overgeneralization of fear to safe stimuli are maladaptive and a major feature of anxiety-related disorders such as post-traumatic stress disorder (PTSD) [2, 3, 4]. However, research data addressing the underlying mechanisms involved in fear generalization at molecular, cellular, and circuit levels remain limited.

Auditory fear conditioning is a robust learning model in which animals rapidly learn to associate a previously neutral tone (the conditioned stimulus, CS) with a coincident aversive stimulus (unconditioned stimulus, US). Re-exposure to the CS alone elicits fear-related responses such as freezing, an index of fear memory. The lateral nucleus of the amygdala (LA) receives input of sensory information and is a key site for association and storage of the auditory cued fear memory [5]. Ghosh and Chattarji [6] identified distinct neuronal populations in the LA of rats that signaled generalized versus cue-specific associations and demonstrated that the same LA neurons that were cue-specific before the occurrence of fear generalization lost their specificity afterwards, thereby tilting the balance of activity toward a greater proportion of generalizing neurons.

In animal models, fear generalization can be modulated by the intensity of the US and strong US (e.g., 0.8 or 1.0 mA footshock) increased fear responses and generalization [7, 8, 9]. Furthermore, single acute restraint stress (RS) prior to conditioning was shown to promote the generalization of contextual fear memory [10]. These notions suggest an advantage of using an experimental model combining prior RS with strong US fear conditioning to induce fear generalization for strong emotional learning under stress. Gastrin-releasing peptide (GRP), a 27-amino acid mammalian neuropeptide, appears to integrate the processing of stress and fear with synaptic plasticity and memory via the G protein-coupled GRP receptor (GRPR) [11]. Notably, accumulating evidence indicates that both GRP and GRPR are highly expressed in the brain regions crucial for stress response and associative fear learning, such as the hypothalamus, anterior pituitary gland, LA, and auditory cortex (AC), playing a role in the strength of fear memory retrieval [12, 13, 14, 15]. Acute RS promotes release of GRP in the amygdala [16]. Recently, Goto et al. [17] found that GRP knockout (KO) mice exhibited an augmented freezing response when they were subjected to fear conditioning that used a mild footshock (0.35 mA) after acute RS. Furthermore, one recent work demonstrated that in the AC, where sensory information is processed and then transmitted to the LA, GRP recruits disinhibitory microcircuits through selective targeting and activation of vasoactive intestinal peptide (VIP)-expressing interneurons [18]. The disinhibition mediated by GRP is crucial for freezing responses to both conditioned stimulus and non-conditioned stimulus but is not relevant to tone discrimination when using moderate footshock (0.6 mA). However, no data have addressed how GRP plays its modulatory role in response to a highly threatening event under stress, with special respect to assessing the risk of experience-related cues.

In the present study, using GRP-KO (*Grp*^−/−^) mice, we first examined the role of GRP in the strength of auditory fear memory and generalization using strong and moderate US fear conditioning protocols that were conducted after a single acute RS. We then investigated how the deletion of the GRP gene or regional administration of GRP into the AC influences auditory fear memory and generalization. Our findings provide a novel insight for understanding the role of GRP in the modulation of associative fear memory acquired following a traumatic event under a stressful situation.

## Materials and methods

### Animals

Animal experiments were approved by the Ethics Committee for Animal Experiments at the University of Toyama (Authorization No. A2021 MED-33) and carried out in accordance with the Guidelines for the Care and Use of Laboratory Animals of the University of Toyama. WT and *Grp*^−/−^ mice were obtained by crossing *Grp*^+/−^ mice maintained in a pure C57BL/6 background, and the genotypes were determined with a polymerase chain reaction (PCR) as described previously [13]. All experiments used 10-week-old male mice, which have normal hearing ability [19, 20]. The mice were kept in a temperature- and humidity-controlled room under a 12 h light/dark cycle (lights on at 7:00 AM) and had ad libitum access to food and water.

### Acute RS

Mice were handed for one week and randomly divided into acute RS and control groups, respectively. The acute RS group mice were placed in an adequately ventilated 50-ml plastic tube (FALCON) for 20 min. They could rotate from a prone to supine position and back again but not turn head to tail. Afterward, they returned to their home cages and rested for 1 h until the fear conditioning began. The control mice were not exposed to RS and were left in their home cages.

### Auditory fear conditioning

Auditory fear conditioning was conducted in a sound-attenuated chamber (CL-M3, O’Hara and Co., Ltd., Japan). Mice were handled and single-housed for 7 days prior to fear conditioning. Two days before fear conditioning, mice were habituated to the conditioning box (context A: 17 × 30 × 10 cm, with transparent walls and a floor of 26 stainless steel rods) for 10 min each day. For fear conditioning, each mouse was placed in the conditioning box for 1 min and then received three pairings of auditory CS (10 kHz, 30 s, 65 dB) co-terminated with US (footshock, 2 s, 0.8 or 0.5 mA) with 1 min inter-pairing intervals. Mice were returned to their home cages 30 s after the last footshock. The conditioning box was cleaned with 75% ethanol before each trail. The auditory fear memory test was conducted 24 h after fear conditioning. Each mouse was placed in a novel box (context B: 10 × 10 × 10 cm, with white walls and a flat floor) for 2 min and then randomly exposed to a conditioned stimulus (CS+, 10 kHz, 2 min, 65 dB) and a non-conditioned stimulus (CS-, 2 kHz, 2 min, 65 dB) with different frequencies from CS+ was unpaired with the US. The novel box was cleaned with 4% acetic acid before each trail. The interval between the two distinct frequency tones was 30 s. For the experiment investigating the responses of CS+-responsive AC neurons to CS-, each mouse was placed in context B and then exposed to CS- only.

Freezing responses were analyzed using Time FZ2 software (O’Hara & Co., Japan). To distinguish better between generalizer and discriminator, we used the discrimination score (DS) obtained by dividing the percentage of freezing in response to the CS+ by that in response to the CS- (CS+/CS-). We applied a DS of 2 to distinguish generalizers (DS < 2) from discriminators (DS > 2) [21].

### Adeno-associated virus (AAV) vector

For constructing the pAAV-c-Fos-rtTA3G plasmid, a DNA fragment containing the *c-fos* promoter, a reverse tetracycline-controlled transactivator (rtTA3G), and a small intron was amplified by PCR and then subcloned into the pAAV-MCS vector (240071, Agilent Technologies, Santa Clara, CA, USA). pAAV-TRE-hM4D(Gi)-mCherry plasmid was constructed by restriction enzyme digest of pAAV-hSyn-hM4D(Gi)-mCherry (Addgene, 50475) with EcoRI and MluI to cut out the reading frame encoding hM4D(Gi)-mCherry and then ligated into EcoRI- and MluI-cut pAAV-TIWB-yellow pre-eGRASP(p32) vector (Addgene, 111582) fragment containing tetracycline responsive element and ITR sequence.

Recombinant AAV vectors were produced using 293 cells (240073, Agilent Technologies) cultured in a 15 cm dish. Cultured cells were maintained in Dulbecco’s Modified Eagle Medium (DMEM, Nacalai Tesque, Kyoto, Japan) supplemented with 10% fetal bovine serum (FBS, Invitrogen, Carlsbad, CA). Approximately 70–80% confluent 293 cells were transfected using a medium containing the constructed expression vector, AAV9 Rep/Cap plasmid (GeneMedi Suzhou Biotechnology, Shanghai, China), and pHelper (240071, Agilent Technologies) mixed with the transfection reagent polyethyleneimine hydrochloride (Polysciences, Inc., Warrington, PA, USA) at a 1:2 ratio (W/V). After 24 h, the transfection medium was discarded, and cells were incubated for another 5 days in an FBS-free DMEM. On day 6, the AAV-containing medium was collected and purified from cell debris using a 0.45 μm Millex-HV syringe filter (SLHV033RS, Merck Millipore, Germany). The filtered medium was concentrated and diluted with PBS three times using the Vivaspin 20 column (VS2041, Sartorius, Germany) after blocking the column membrane with 1% bovine serum albumin (Invitrogen) in PBS. The viral titers were determined by qRT-PCR. The average titters of AAV-TRE-hM4D(Gi)-mCherry and AAV-c-fos-rtTA3G were 1.4 × 10^12^ and 1.21 × 10^13^ vector genomes (v.g.) /ml, respectively.

### Surgery

For GRP infusion experiments, six-week-old male mice were anesthetized with an anesthetic combination of 0.3 mg/kg medetomidine, 4.0 mg/kg midazolam and 5.0 mg/kg butorphanol (intraperitoneal injection) and placed in a stereotactic frame (Narishige, Tokyo, Japan). An incision was made in the center of the scalp and 26-gauge bilateral guide cannulas were implanted into AC (AP: -2.7 mm; ML: 4.4 mm; DV: -3.3 mm relative to the bregma). Guide cannulas were fixed with two anchor screws and cement on the skull. Mice recovered from the surgery for at least 10 days before behavioral experiments started. GRP (50 µg/ml, 0.5 µl/side, Phoenix Pharmaceuticals Inc., USA) or saline (0.9% w/v, 0.5 µl/side) was infused into the AC of RS-exposed mice through the injection needle at a 0.5 µl/min rate. The needle was pulled out 2 min after the infusion to ensure drug absorption. To confirm the injection site, we injected BODIPY™ TMR-X conjugated muscimol (Invitrogen) through guide cannula immediately after the completion of behavioral tests.

For AAV injection experiments, six-week-old male mice were deeply anesthetized as described above and placed in the stereotactic frame. A mixture of AAV-c-Fos-rtTA3G and AAV-TRE-hM4D(Gi)-mCherry (1 µl/side) was bilaterally injected into the AC (AP: -2.7 mm; ML: 4.4 mm; DV: -3.3 mm) using 33-gauge needles with Hamilton syringe at a 0.1 µl/min rate and pulled out 5 min later. Mice were allowed to recover in their home cages for 4 weeks before behavioral experiments.

### Immunofluorescent staining

Mice were anesthetized with intraperitoneal injection of isozol (100 mg/kg, Nichi-Iko Pharmaceutical Co., Toyama, Japan) 1.5 h after fear memory tests and then transcardially perfused with phosphate-buffered saline (PBS, pH 7.4) followed by 4% ice-cold paraformaldehyde (PFA) in 0.1 M phosphate buffer (PB). Brains were post-fixed in PFA for overnight at 4 °C, and then immersed in 0.1 M PB containing 30% sucrose for 36 h at 4 °C. The brains were cut into 25-µm-thick sections with a frozen microtome (Leica CM1850, Lecia, Germany). On the first day of staining, the brain sections were washed with PBS three times and blocked with Protein Block Serum-Free (DAKO Cytomation, CA) for 10 min at room temperature (RT). Then the sections were incubated with anti-c-Fos antibody (1: 1000, Cat# ABE457, RRID: AB_2631318, EMD Millipore Corp., USA) overnight at 4 °C. On the following day, the sections were washed with PBS three times and then incubated with Alexa Fluor-488 conjugated donkey anti-rabbit antibody (1:500, Cat# A21206, RRID: AB_2535792, Invitrogen USA) for 1 h at RT. Then, the sections were washed with PBS and adhered to glass slides. One drop of *SlowFade* Gold antifade reagent (Invitrogen, USA) was added onto the slide, and then coverslips were placed.

Images were taken using a fluorescence microscope (KEYENCE BZX-800, KEYENCE, Japan). Two sections per mouse from the middle region of the virus injection sites, located between bregma − 2.69 mm and − 3.27 mm, with evenly distributed mCherry + neurons were used to quantify mCherry-positive and c-Fos-positive cells in the AC (secondary auditory cortex and auditory association cortex). All sections were counted by us who were blind to the genotype of the mice.

### Statistical analysis

Statistical analysis was performed using GraphPad Prism 9 software (GraphPad Software Inc., San Diego, CA, USA) and Excel Statistics (Statcel 2; Social Survey Research Information Co. Ltd., Tokyo, Japan). Prior to any statistical analysis, the normality of data was evaluated using the Shapiro-Wilk test. Three-way repeated measures ANOVA was used to assess the freezing levels in mice with or without acute RS during fear conditioning. Two-way repeated-measures ANOVA was used to assess the freezing levels between saline and GRP treatments during fear conditioning. One-way ANOVA was used for analyzing freezing levels during the tests consisting of multiple groups and Student’s *t*-test was used for the comparison of two groups. The number of mCherry- and c-Fos-positive neurons in the AC was analyzed using one-way ANOVA. Following significant ANOVAs, Tukey’s multiple comparisons tests were used. Fisher’s exact test was used to evaluate the differences in percentages of generalization between groups. All experimental data are expressed as means ± standard error of the mean (S.E.M.) and the level of statistical significance was defined as *p* < 0.05.

Mice were randomly assigned to experimental groups prior to experimentation. No statistical methods were used to pre-determine sample sizes, but our sample sizes are similar to those generally employed in the field. Animals with viral injections off target or damaged during the experiment were excluded from the statistical analysis.

## Results

### GRP promotes the fear response to CS+ and CS- after acute RS exposure

**Fig. 1:**
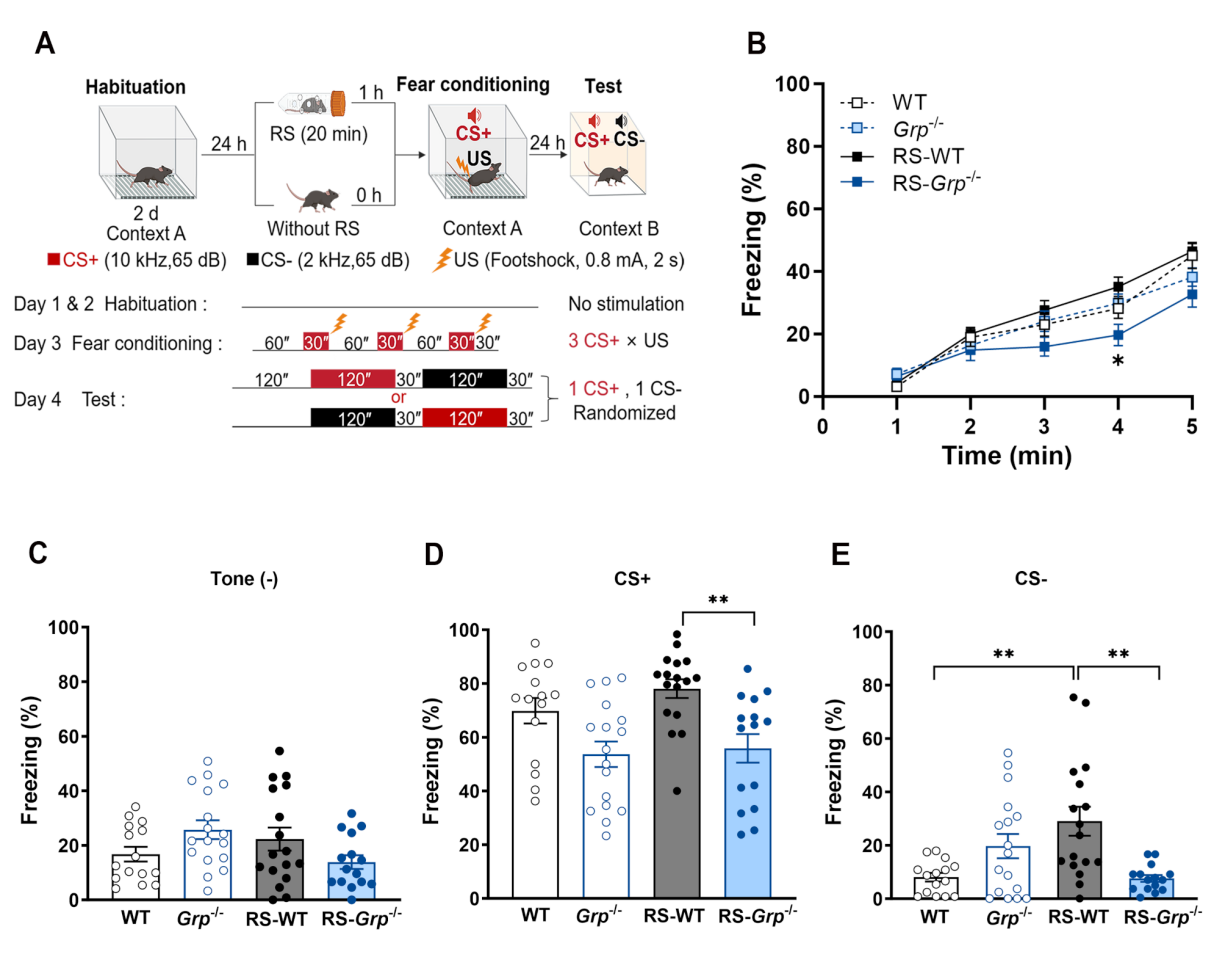
GRP is required for enhancing fear responses to CS+ and CS- after acute RS exposure. **(A)** Experimental schedule for habituation, RS exposure, fear conditioning, and memory test. After acclimating to the training context for two consecutive days, WT and *Grp*^−/−^ mice underwent strong auditory fear conditioning with or without prior RS exposure. The conditioning protocol included three CS+ × US (0.8 mA footshock) parings, followed by testing fear responses to CS+ and CS- 24 h post-conditioning. **(B)** RS-WT (*n* = 17) mice exhibited significantly higher freezing levels compared to RS-*Grp*^−/−^ mice (*n* = 15) during fear conditioning. Three-way repeated measures ANOVA revealed a significant effect of genotype, F_(1, 60)_ = 5.403, *p* = 0.024, and an interaction effect of RS × genotype, F_(1, 60)_ = 4.189, *p* = 0.045. Tukey’s multiple comparisons test, ^*^p < 0.05. **(C-E)** The freezing levels during the memory test. **(C)** No significant differences were observed in freezing levels without tone presentation across experimental groups. **(D)** RS-WT mice exhibited significantly higher freezing responses to CS+ than RS-*Grp*^−/−^ mice (One-way ANOVA, F(3,60) = 6.639, *p* < 0.01; Tukey’s multiple comparisons test, ^**^p < 0.01). **(E)** RS-WT mice showed significantly greater freezing responses to CS- compared to WT (*n* = 15) and RS-*Grp*^−/−^ mice (One-way ANOVA, F(3,60) = 7.012, *p* < 0.01; Tukey’s multiple comparisons test, ^**^p < 0.01). Individual values are represented by circles. Data are presented as the means ± SEM. Without tone presentation: tone (-); WT: wild-type mice, RS-WT: wild-type mice with prior RS exposure, *Grp*^−/−^: GRPKO mice, RS-*Grp*^−/−^: GRPKO mice with prior RS exposure

We first investigated the role of GRP in mediating the effect of prior stress on auditory fear conditioning and fear generalization using wild-type (WT) and *Grp*^−/−^ mice. Mice underwent fear conditioning using three pairings of conditioned stimulus (CS+, 10 kHz tone) and high-intensity footshock (0.8 mA). This protocol was conducted either with or without prior exposure to RS. Freezing responses to CS+ and the non-conditioned tone (CS-, 2 kHz tone) were assessed 24 h post-conditioning (Fig. 1A). During fear conditioning, WT and *Grp*^−/−^ mice exhibited comparable freezing responses to footshocks (Fig. 1B). However, RS-*Grp*^−/−^ mice displayed significantly lower freezing levels than RS-WT mice during conditioning (Fig. 1B, *p* < 0.05, three-way repeated measures ANOVA with Tukey’s multiple comparisons test). These results suggest that GRP is essential for associating CS+ with US after RS exposure. In the memory test, all experimental groups demonstrated similar freezing levels without tone presentation (Fig. 1C). Upon CS+ presentation, RS-WT mice showed significantly higher freezing than RS-*Grp*^−/−^ mice (Fig. 1D, *p* < 0.01, one-way ANOVA with Tukey’s multiple comparisons test). Additionally, RS-WT mice exhibited significantly higher freezing responses to CS- compared to WT and RS-*Grp*^−/−^ mice (Fig. 1E, *p* < 0.01, one-way ANOVA with Tukey’s multiple comparisons test).

A previous study demonstrated that *Grp*^−/−^ mice exhibited enhanced fear responses to CS+ compared to WT mice when low-intensity footshocks (0.35 mA) were used during fear conditioning [17]. To investigate whether the strength of the US influences GRP’s role in modulating fear responses to CS+, we conducted auditory fear conditioning using a moderate-intensity footshock (0.5 mA) protocol (Supplementary Fig. 1A). All experimental groups showed similar levels of freezing during both fear conditioning (Supplementary Fig. 1B) and memory tests (Supplementary Figs. 1 C-E). These results suggest that GRP is specifically required for enhancing fear response to both CS+ and CS- in conditioning involving strong US after acute RS exposure.

### GRP is required for the facilitatory effects of acute RS on fear generalization

**Fig. 2:**
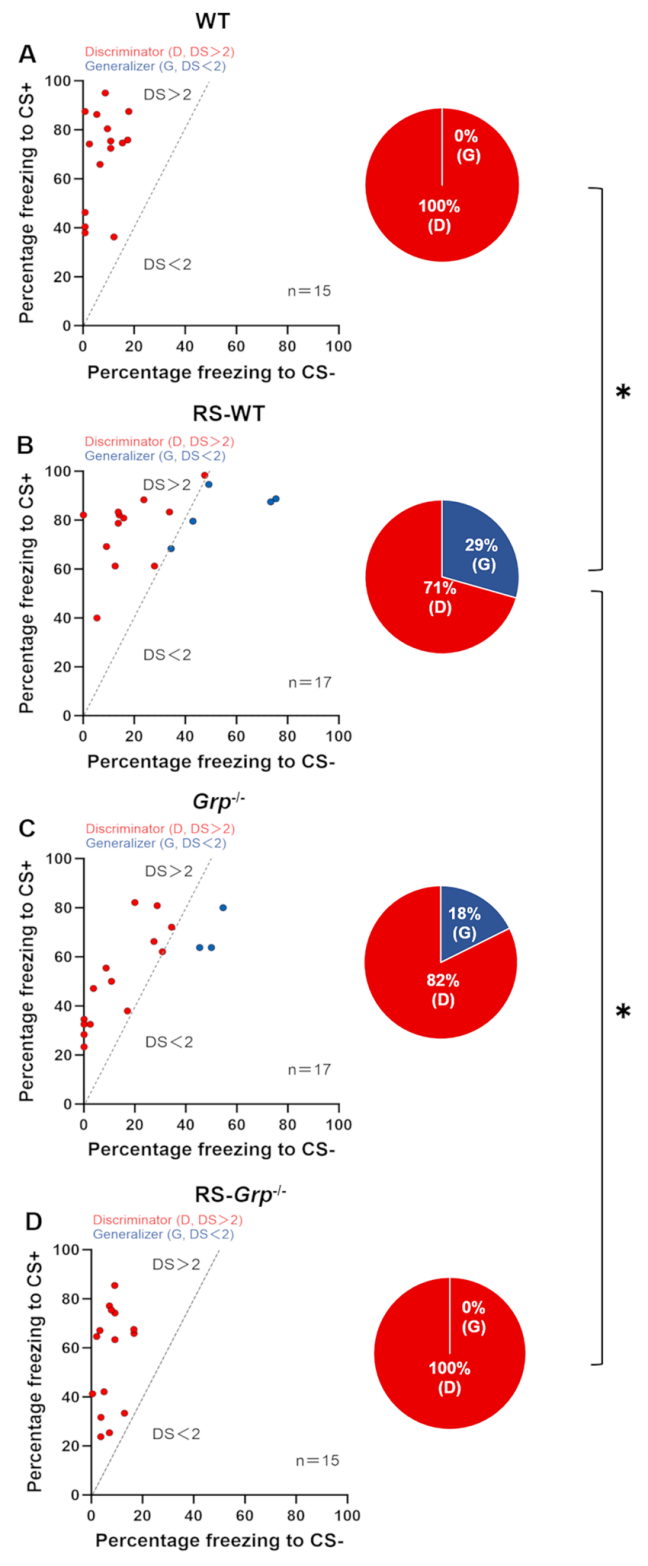
GRP is required for auditory fear generalization after acute RS exposure. **(A-D**,** left)**: Scatter plots illustrate freezing responses of each WT (*n* = 15), *Grp*^−/−^ (*n* = 17), RS-WT (*n* = 17), and RS-*Grp*^−/−^ (*n* = 15) mice to CS+ and CS-, respectively. A dotted line marks a discrimination score (DS) of 2. Mice with a DS greater than 2 are represented by red circles (discriminators), while those with a DS less than 2 are marked with blue circles (generalizers). **(A-D**,** right)**: Pie charts show the proportion of discriminators (red) and generalizers (blue) in WT, *Grp*^−/−^, RS-WT, and RS-*Grp*^−/−^ mice, with percentages represented in the charts. Fear generalization was significantly higher in RS-WT mice compared to WT and RS-*Grp*^−/−^ mice (Fisher’s exact test, ^*^p < 0.05). WT: wild-type mice, RS-WT: wild-type mice with prior RS exposure, *Grp*^−/−^: GRPKO mice, RS-*Grp*^−/−^: GRPKO mice with prior RS exposure

Having found that GRP is involved in enhancing the fear response to both CS+ and CS- in strong US conditioning after acute RS exposure, we then investigated the role of GRP in distinguishing between CS+ and CS- in the conditioning with or without prior RS exposure. To assess the extent to which animals differentiated or generalized between the two auditory stimuli, we defined a Discrimination Score (DS) by dividing the percentage of freezing in response to CS+ by that in response to the CS- (CS+/CS-). We applied a DS of 2 to distinguish generalizers (G, DS < 2) from discriminators (D, DS > 2) as reported [21]. Using this criterion, we found that all WT mice were discriminators (Fig. 2A), while 3 of 17 (18%) *Grp*^−/−^ mice exhibited generalized fear (Fig. 2C). However, the percentage of fear generalization did not significantly differ between WT and *Grp*^−/−^ mice. Among the mice with prior RS exposure, 5 of 17 (29%) RS-WT mice exhibited generalized fear (Fig. 2B) and none of the RS-*Grp*^−/−^ mice exhibited fear generalization (Fig. 2D). The percentage of fear generalization was significantly higher in RS-WT mice compared to WT and RS-*Grp*^−/−^ mice (*p* < 0.05, Fisher’s exact test). These results suggest that GRP is required for prior RS-induced enhancement in fear generalization.

### Infusion of GRP into the AC of *Grp*^−/−^ mice restores fear response to CS- and generalization after acute RS

**Fig. 3:**
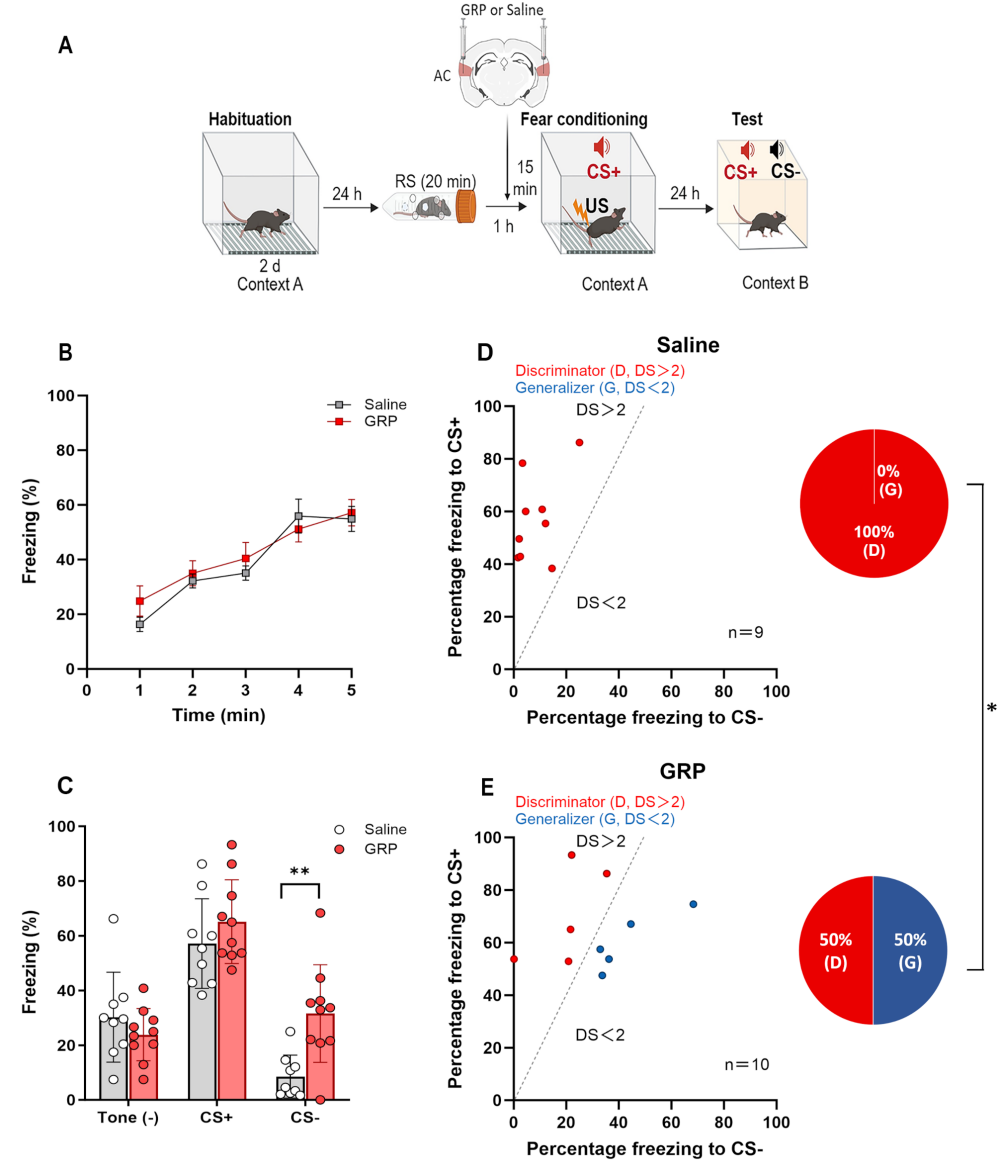
Infusion of GRP into the AC of RS-*Grp*^−/−^mice restores fear response to CS- and generalization after prior RS exposure. **(A)** Experimental schedule for habituation, RS exposure, drug infusion, fear conditioning, and memory test. *Grp*^−/−^ mice were acclimated to the training context for two consecutive days. On the third day, they underwent a 20-min acute RS session before being returned to their home cages. GRP (50 µg/ml, 0.5 µl) or saline was infused into the AC for 15 min prior to fear conditioning. The mice were then conditioned using three CS+ × US (0.8 mA footshock) pairings, and their fear responses to CS+ and CS- were tested 24 h after conditioning. **(B)** GRP infusion into the AC did not alter freezing levels during fear conditioning. **(C)** Compared with saline-treated mice (*n* = 9), GRP-treated mice (*n* = 10) exhibited significantly higher levels of freezing in response to CS- but not to tone (-) or CS+ (Student’s *t*-test, ^**^p < 0.01). Data are presented as the means ± SEM. **(D and E**,** left)**: Scatter plots illustrate freezing responses of saline- and GRP-treated *Grp*^−/−^ mice to CS+ and CS-, respectively. Red and blue circles indicate mice classified as discriminators (DS > 2) and generalizers (DS < 2), respectively. **(D and E**,** right)**: Pie charts depict the proportion of discriminators (red) and generalizers (blue) in saline- and GRP-treated *Grp*^−/−^ mice. GRP-treated mice showed a significantly higher percentage of generalization compared to saline-treated mice (Fisher’s exact test, ^*^p < 0.05)

The GRP-mediated disinhibition of AC microcircuits is crucial for freezing responses to both CS+ and CS- [18]. Therefore, we investigated whether regional administration of GRP into the AC of *Grp*^−/−^mice would influence the fear responses to CS+ and CS-, as well as fear generalization after acute RS exposure. *Grp*^−/−^ mice were subjected to 20-min of RS and were fear conditioned 1 h later. Saline or GRP (50 µg/ml, 0.5 µl) was infused into AC 15 min before fear conditioning (Fig. 3A). During the fear conditioning, there was no significant difference in freezing levels between the GRP- and saline-treated mice (Fig. 3B). The GRP-treated mice exhibited significantly higher levels of freezing to CS- compared to saline-treated mice during the tests (Fig. 3C, *p* < 0.01, Student’s *t*-test). In contrast, freezing levels were comparable between GRP- and saline-treated mice during tests without tone presentation or with CS+ presentation (Fig. 3C). Consistent with the result described above, all the saline-treated RS-*Grp*^−/−^ mice were discriminators (Fig. 3D). In contrast, 5 of 10 (50%) GRP-treated RS-*Grp*^−/−^ mice exhibited generalized fear (Fig. 3E). The percentage of generalization was significantly higher in GRP-treated mice compared to the saline-treated mice (*p* < 0.05, Fisher’s exact test). These results suggest that GRP in the AC is essential for enhancing the fear responses to CS- and facilitating auditory fear generalization in conditioning involving strong US after acute RS exposure.

### GRP contributes to the generalization of the CS+ responsive neurons to respond to CS- after acute RS exposure

**Fig. 4:**
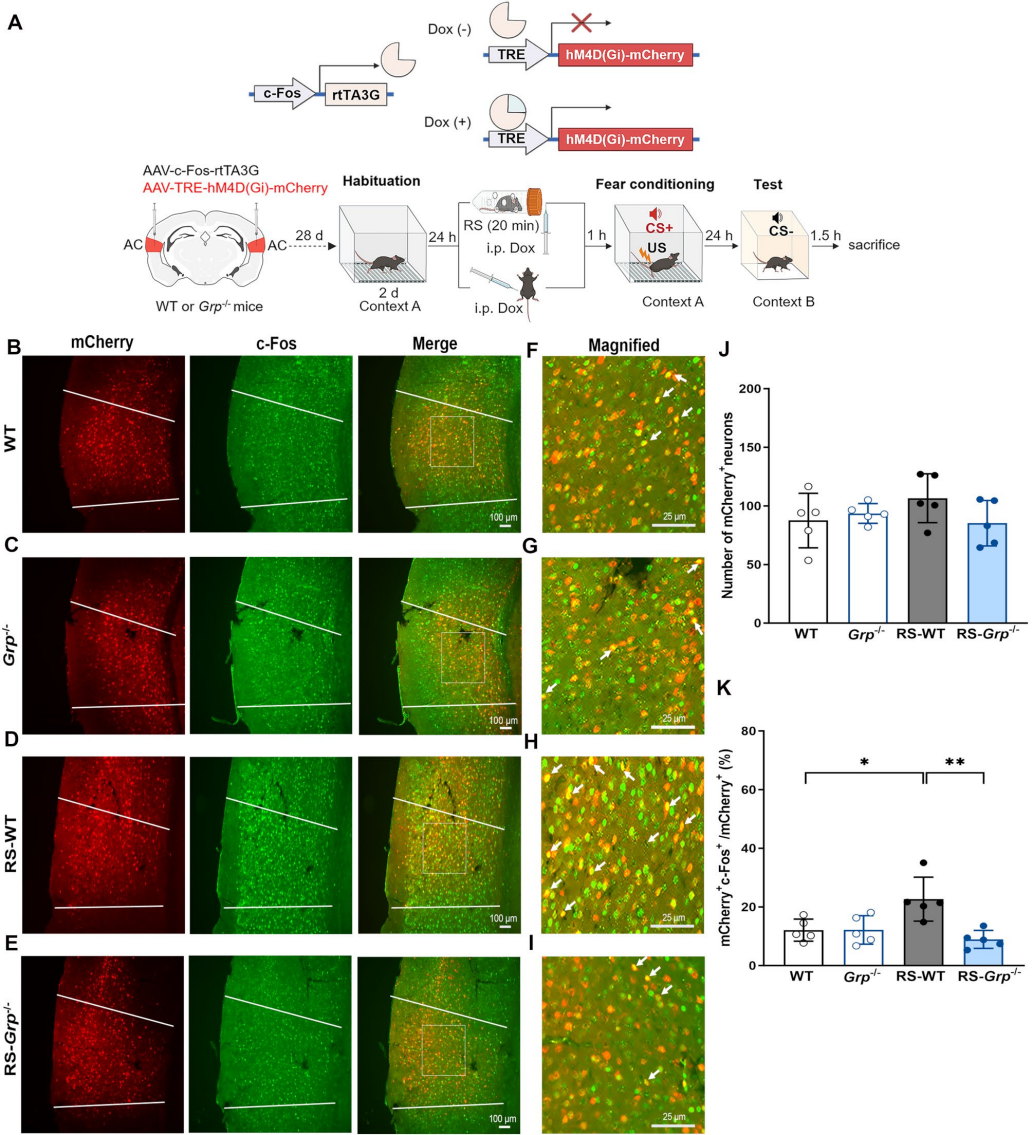
GRP is essential for CS+-responsive neurons within the AC to respond to CS- after acute RS exposure. **(A)** Top: Experimental design to label CS+-responsive neurons during fear conditioning using mCherry. Bottom: Experimental schedule includes habituation, RS exposure, doxycycline (Dox) injection, fear conditioning, and memory test. Four weeks after AAV injection, WT and *Grp*^−/−^ mice acclimated to the training context for two consecutive days were subjected to auditory fear conditioning with or without prior RS exposure. Dox was administered 1 h before fear conditioning. Mice were conditioned using three CS+ × US (0.8 mA) pairings, and fear responses to CS- were tested 24 h later. Mice were sacrificed 90 min following the fear memory test. **(B-E)** Representative images display mCherry^+^ and c-Fos^+^ immunofluorescence in the AC of WT, *Grp*^−/−^, RS-WT, and RS-*Grp*^−/−^ mice. Scale bars = 100 μm (*n* = 5). **(F-I)** Magnified images highlight neurons co-expressing c-Fos and mCherry within square areas (indicated by white arrows). Scale bars = 25 μm. **(J)** No significant differences in the number of mCherry^+^ neurons among WT, *Grp*^−/−^, RS-WT, and RS-*Grp*^−/−^ mice. **(K)** RS-WT mice exhibited significantly higher proportions of c-Fos^+^ positive cells among mCherry^+^ cells compared to WT and RS-*Grp*^−/−^ mice (One-way ANOVA, F_(3,16)_ = 6.980, *p* < 0.01; Tukey’s multiple comparisons test, ^*^p < 0.05, ^**^p < 0.01). Data are presented as means ± SEM. WT: wild-type mice; RS-WT: wild-type mice with prior RS exposure; *Grp*^−/−^: GRPKO mice; RS-*Grp*^−/−^: GRPKO mice with prior RS exposure

Observing that RS-WT mice showed reduced discrimination between CS+ and CS-, we aimed to investigate whether AC neurons responsive to CS+ during fear conditioning would also be reactivated by the CS- during the test. To label the CS+ responsive neurons, we injected the mixture of AAVs into the AC, expressing reverse tetracycline-controlled transactivator (rtTA) under the control of the *c-fos* promoter and a hM4Di red fluorescent protein (hM4Di-mCherry) under the control of the tetracycline-responsive element (TRE). Four weeks after AAV injection, WT and *Grp*^−/−^ mice were subjected to strong fear conditioning, either with or without prior exposure to RS. Doxycycline (Dox) was injected immediately after RS exposure to label CS+-related AC neurons activated during the fear conditioning. The fear responses to CS- were tested 24 h after fear conditioning (Fig. 4A). Mice were sacrificed 90 min after tests and c-fos staining of brain sections were conducted. The number of CS+-responsive neurons activated during fear conditioning (mCherry^+^) was comparable across WT and *Grp*^−/−^ mice, either with or without acute RS exposure (Figs. 4B-J). We then evaluated the percentage of neurons in the AC that were initially responsive to the CS+ during the fear conditioning (mCherry^+^) and subsequently activated by CS- during the test (c-Fos^+^). The percentage of CS+-responsive neurons reactivated to CS- (mCherry^+^ c-Fos^+^/mCherry^+^) was significantly higher in RS-WT mice compared to WT and RS-*Grp*^−/−^ mice (Fig. 4K, RS-WT vs. WT, *p* < 0.05; RS-WT vs. RS-*Grp*^−/−^, *p* < 0.01; one-way ANOVA with Tukey’s multiple comparisons test). To investigate whether the CS+-responsive neurons in the AC are more likely to react to the CS-, we assessed the numbers of mCherry^+^ c-Fos^+^ and mCherry^−^ c-Fos^+^ cells in WT, RS-WT, and RS-*Grp*^−/−^ mice. Our results indicate that the number of mCherry^+^ c-Fos^+^ cells in RS-WT mice was significantly higher than in WT and RS-*Grp*^−/−^ mice (Supplementary Fig. 2A, RS-WT vs. WT, *p* < 0.05; RS-WT vs. RS-*Grp*^−/−^, *p* < 0.01; one-way ANOVA with Tukey’s multiple comparisons test). In contrast, the number of mCherry^−^ c-Fos^+^ cells are comparable across the three groups of mice (Supplementary Fig. 2B). These findings suggest that GRP is necessary for fear generalization, as it enables CS+ responsive neurons to shift and respond to CS- in conditioning involving strong US after acute RS exposure.

## Discussion

In our experimental model using strong US auditory fear conditioning conducted following single acute RS, designed for inducing fear generalization, we investigated the role of GRP in fear responses to CS+ and CS-. We found that GRP is required for the strength of fear behavior in response to CS+ and the fear generalization to CS-. We further present that in the same experiment model, GRP in the AC is essential for generalization of CS+ responsive neurons to respond to CS- during fear memory retrieval. Our findings provide the first evidence of a neuromodulator, GRP, involved in the mechanisms underlying the occurrence of maladaptive fear under stressful situations and offer a novel target for developing new strategies for treating anxiety-related disorders.

Unlike one recent report that identified GRP as an inhibitory neuromodulator for controlling strength of fear memory induced by prior RS exposure [17], we found that this peptide is required for the strengthening of the fear memory in strong US conditioning after acute RS exposure. This was demonstrated by RS-*Grp*^−/−^ mice exhibiting significantly lower levels of freezing responses to CS+ compared to RS-WT mice during memory tests (Fig. 1D). To investigate whether the strength of the US influences GRP’s role in regulating fear responses to CS+, we conducted auditory fear conditioning using a moderate-intensity footshock (0.5 mA) protocol, and found that across all experimental groups, the freezing levels were equivalent during fear conditioning (Supplementary Fig. 1B) and memory tests (Supplementary Figs. 1 C-E). Together with the previously published data from fear conditioning experiments using low-intensity US (i.e., 0.35 mA) following RS exposure [17], our results suggest that GRP’s role in modulating or facilitating fear responses to CS+ may be influenced by the intensity of the US. Although direct laboratory data is lacking, several lines of evidence from previous works help understand the enhancing effect of GRP on fear memory and generalization in our experimental model. First, either previous stressful exposure or strong US fear conditioning has been shown to evoke a high level of corticosterone release [9], which promotes GRP release in the central nucleus of the amygdala and prefrontal cortex [22, 23]. Second, the elevation of GRP in the rat amygdala by exposure to a shock might stimulate the release of adrenocorticotropic hormone [24], which promotes the synthesis of corticosterone from the cholesterol and its release from the adrenal cortex. These findings suggest that GRP and corticosterone reciprocally regulate their release levels and coordinately act to enhance fear response in highly stressful and threatening situations.

GRP is highly expressed in the LA, a key structure for association and storage of the auditory fear memory [5], and the cortical regions that project to the LA, such as the AC and medial geniculate nucleus [13, 15]. During auditory fear conditioning, auditory information reaches LA through two main pathways: directly from the medial geniculate nucleus or indirectly via the AC [25, 26]. The AC refines and enhances the auditory signals before they reach the amygdala, contributing to the precision of fear responses [26, 27]. When a sound is paired with an aversive event, the AC performs a detailed analysis of the physical properties of this sound, encoding and storing them for a long time [27, 28]. In our assessment using the DS criterion, we demonstrated that the percentages of generalization after acute RS are significantly higher in RS-WT mice compared to RS-*Grp*^−/−^ mice, suggesting that GRP mediates the enhancing effect of prior acute stress on fear generalization when confronted with a subsequent strong aversive event. This result prompted us to examine the influence of GRP infusion into the AC of *Grp*^−/−^mice on fear responses to CS+ and CS-, as well as fear generalization after acute RS exposure. We found that the GRP infusion restores the freezing behavior of RS-*Grp*^−/−^ mice in response to CS- and the extent of fear generalization assessed by the DS method. Additionally, we demonstrated that GRP is required for generalization of CS+-responsive neurons within the AC to respond to CS- after acute RS exposure.

It is noteworthy that there is no significant difference in the percentage of generalizer between the WT and *Grp*^−/−^ mice without prior RS exposure. This result aligns with other works showing that neither genetic deletion of GRPR nor pharmacological inactivation of the AC affects the extent of fear generalization in fear conditioning models without prior stress exposure, suggesting that GRP primarily serves to mediate the enhancing effect of previous stress on fear generalization. Previous studies have demonstrated that the gamma-aminobutyric acid (GABA) ergic system is a key component in modulating emotional reactions to stressful stimuli. Single RS exposure reportedly elicits hyperexcitability of basolateral amygdala (BLA) neurons accompanied by depressed GABAergic inhibition (disinhibition) [29]. Administration of bicuculine, an antagonist of the GABA-A site, into BLA before fear conditioning emulated the promoting effect of prior RS on contextual fear generalization, whereas administration of midazolam, which increases the inhibitory activity through the GABA-A receptor, decreased the fear generalization [10].

Recently, Melzer et al. [18] demonstrated that GRP recruits disinhibitory cortical microcircuits through selective targeting and activation of VIP-expressing neurons in many cortical regions, suggesting a role for GRP in facilitating excitability of the glutamatergic neurons. The VIP cells-dependent disinhibitory mechanism for excitatory neurons has also been identified in BLA [30]. We thus speculate that GRP is likely involved in mediating VIP cells-dependent disinhibition of excitatory neurons in these brain regions when encountering a threatening event in highly stressful situations, thereby promoting fear generalization. Further studies are required to clarify their relationship.

In addition, the use of male animals in this study, which limit our understanding of sex disparity in fear generalization. Studies in humans have shown that the prevalence of PTSD in women is twice that in men following a traumatic experience [31], so the relationship between sex differences and the role of GRP in auditory fear generalization under highly stressful situations is worth further investigation.

In summary, we report the facilitatory role of GRP in enhancing fear response and generalization induced in our experimental model of strong US fear conditioning after acute prior RS. GRP’s action on generalization of the CS+-responsive AC neurons to respond to CS- is distinct from its suppressive effect on fear response induced in weak-US fear conditioning with acute prior RS [17]. Further studies are needed to confirm the facilitatory action of GRP/GRPR signaling in remote fear memories learned in highly stressful and threatening situations. Our findings suggest GRP as a novel target for developing new strategies for treating anxiety-related disorders.

## Electronic Supplementary Material

Below is the link to the electronic supplementary material.

Supplementary Material 1

## Data Availability

No datasets were generated or analysed during the current study.

## Abbreviations

GRP: Gastrin-releasing peptide
GRPR: GRP receptor
CS+: Conditioned stimulus
CS-: Non-conditioned stimulus
US: Unconditioned stimulus
RS: Restraint stress
AC: Auditory cortex
LA: Lateral nucleus of the amygdala
BLA: Basolateral amygdala
GABA: Gamma-aminobutyric acid
VIP: Vasoactive intestinal peptide
Dox: Doxycycline
PTSD: Post-traumatic stress disorder
ANOVA: Analysis of variance
