# Supplementary material for: Gastrin-releasing peptide is essential for generalization of auditory conditioned fear under stress
Source: Mol Brain. 2025 May 15;18:44. doi: 10.1186/s13041-025-01214-w (PMC12080023; doi:10.1186/s13041-025-01214-w)
Supplement: Supplementary file 1 — Supplementary Material 1 [file 13041_2025_1214_MOESM1_ESM.docx]

**
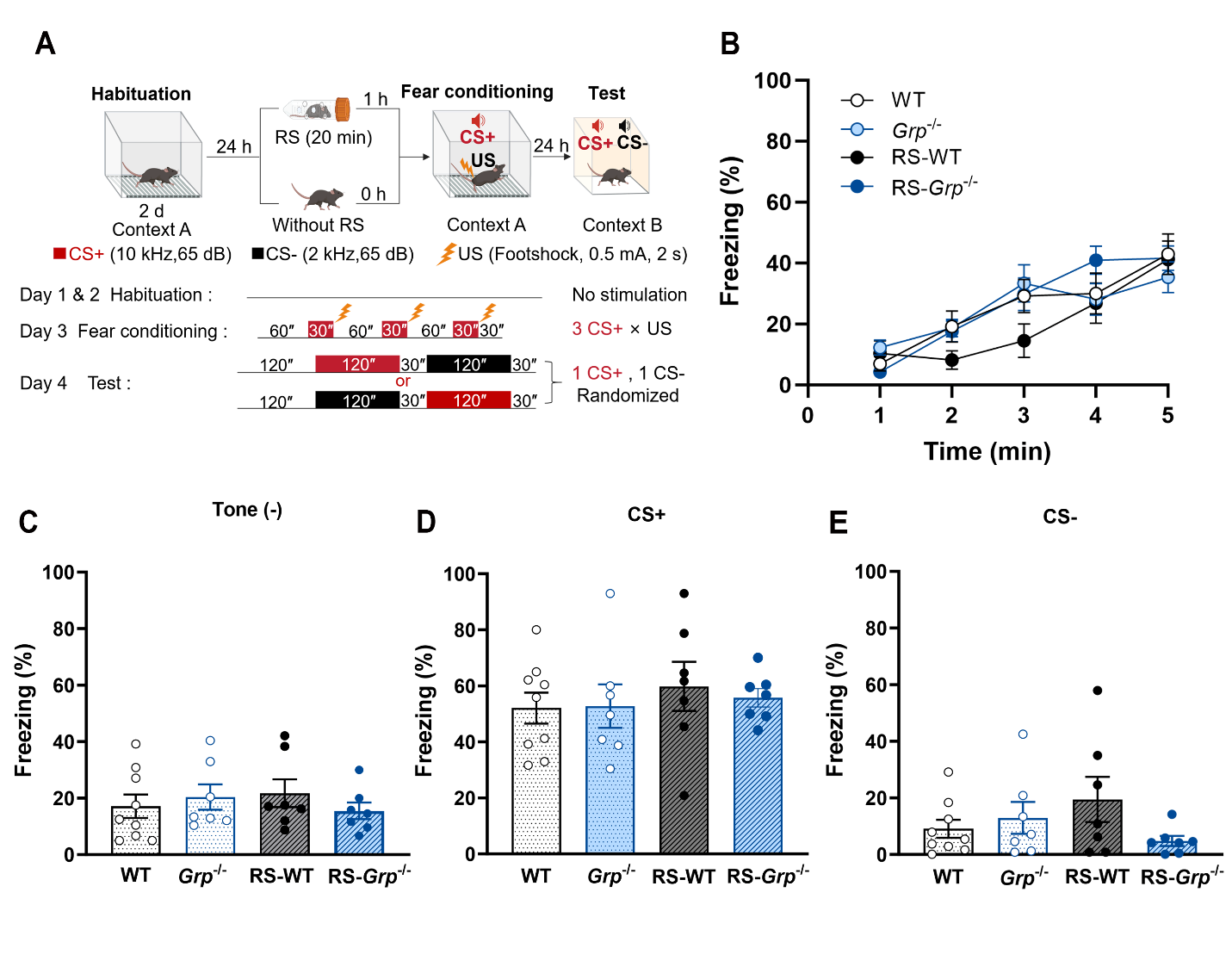
**

**Supplementary figure 1. GRP deletion does not affect fear responses to CS+ or CS- under moderate auditory fear conditioning with or without prior restraint stress**

**(A)** Experimental schedule for habituation, RS exposure, fear conditioning, and memory test. After acclimating to the training context for two consecutive days, WT and *Grp*^-/-^ mice underwent moderate auditory fear conditioning with or without prior RS exposure. Conditioning involved three CS+ × US (0.5 mA footshock) pairing protocols, followed by a fear response test 24 h later. **(B)** No significant differences in freezing levels were observed between WT and *Grp*^-/-^ mice, regardless of stress exposure. **(C-E)** Freezing responses to tone (-), CS+, and CS- revealed no significant differences among WT (n = 9), *Grp*^-/-^ (n = 7), RS-WT (n = 7), and RS-*Grp*^-/-^ (n = 7) groups. Individual data points are displayed as circles, with values represented as means ± SEM. WT: wild-type mice; RS-WT: wild-type mice with prior RS exposure; *Grp*^-/-^: GRPKO mice; RS-*Grp*^-/-^: GRPKO mice with prior RS exposure.

**
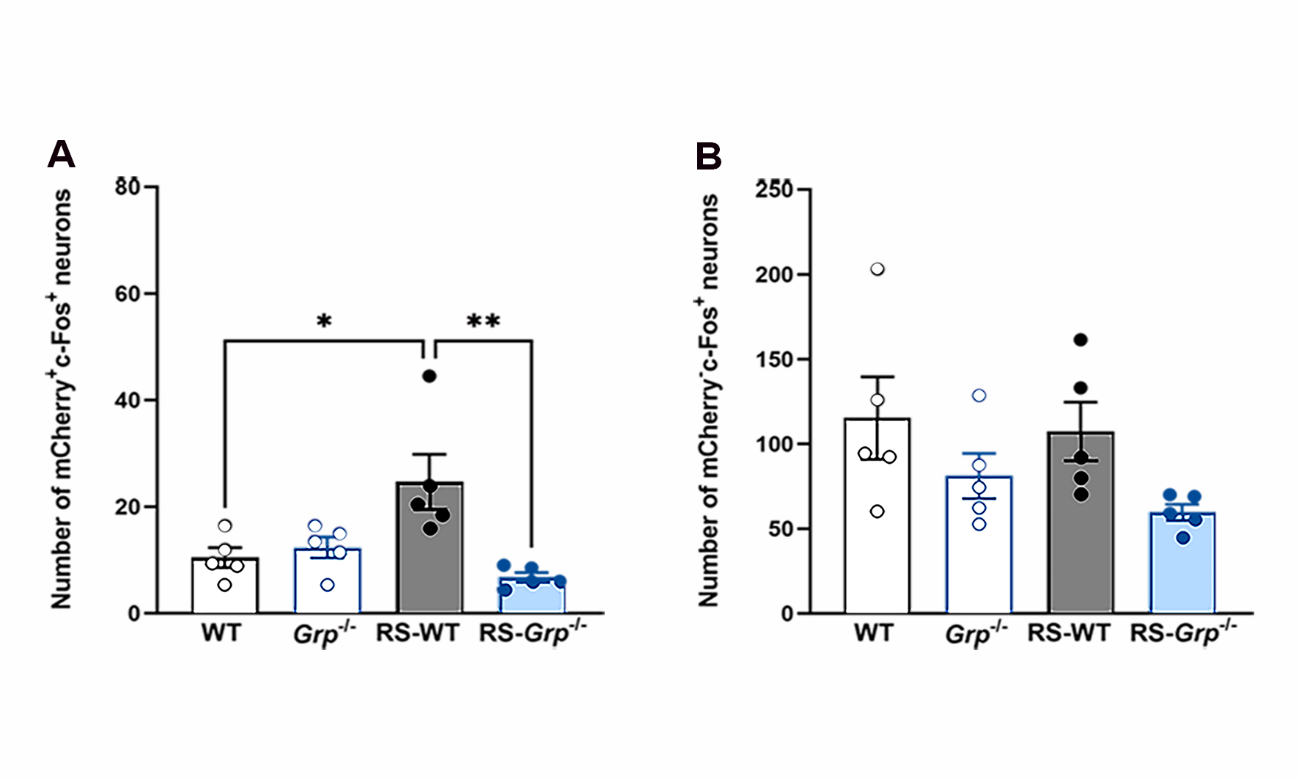
**

**Supplementary figure 2. GRP makes CS+-responsive neurons more likely to shift and respond to CS- in strong auditory fear conditioning following acute RS exposure.**

**(A)** RS-WT mice exhibited significantly higher number of mCherry^+^ c-Fos^+^ cells compared to WT and RS-*Grp*^-/-^ mice (One-way ANOVA, F_(3,16)_ = 7.096, p < 0.01; Tukey’s multiple comparisons test, ^⁎^p < 0.05, ^⁎⁎^p < 0.01). **(B)** The number of mCherry^-^ c-Fos^+^ cells was comparable across the WT, *Grp*^-/-^, RS-WT, and RS-*Grp*^-/-^ mice. Data are presented as means ± SEM (n = 5 per group). WT: wild-type mice; RS-WT: wild-type mice with prior RS exposure; *Grp*^-/-^: GRPKO mice; RS-*Grp*^-/-^: GRPKO mice with prior RS exposure.
